# CPNE7-derived peptides CDP4 and UG28 as pro-angiogenic factors for endothelial regeneration

**DOI:** 10.3389/fphar.2026.1839788

**Published:** 2026-09-07

**Authors:** Agata Tymińska, Magdalena Narajczyk, Aneta Skoniecka, Natalia Karska, Milena Deptuła-Knioła, Szymon Mania, Karolina Kondej, Piotr M. Skowron, Susanna Miettinen, Sylwia Rodziewicz-Motowidło, Michał Pikuła

**Affiliations:** 1 Laboratory of Tissue Engineering and Regenerative Medicine, Division of Embryology, Department of Anatomy, Faculty of Medicine, Medical University of Gdańsk, Gdańsk, Poland; 2 Bioimaging Laboratory, Faculty of Biology, University of Gdańsk, Gdańsk, Poland; 3 Department of Biomedical Chemistry, Faculty of Chemistry, University of Gdańsk, Gdańsk, Poland; 4 Department of Chemistry, Technology and Biotechnology of Food Gdańsk University of Technology, Gdańsk, Poland; 5 Department of Plastic Surgery, Medical University of Gdańsk, Gdańsk, Poland; 6 Department of Surgery and Urology for Children and Adolescents, Faculty of Medicine, Medical University of Gdańsk, Gdańsk, Poland; 7 Department of Molecular Biotechnology, University of Gdańsk, Gdańsk, Poland; 8 Adult Stem Cell Group, Faculty of Medicine and Health Technology, Tampere University, Tampere, Finland; 9 Tays Research Services, Wellbeing Services County of Pirkanmaa, Tampere University Hospital, Tampere, Finland; 10 Department of Biochemistry, University of Physical Education and Sport, Gdańsk, Poland

**Keywords:** angiogenic agents, biomaterial constructs, endothelial cells, peptides, regenerative medicine and tissue engineering

## Abstract

Background and purpose. Efficient endothelial regeneration is fundamental to microcirculatory restoration and hard tissue repair. CPNE7 family peptides have previously been shown to regulate osteogenesis and chondrogenesis, however, their involvement in vascular reconstruction has not been explored. Experimental approach. In this study, we investigated the effect of two peptides derived from CPNE7, CDP4 and UG28, on human endothelial cells *in vitro*, evaluating their influence on proliferation, migration, chemotaxis, capillary-like structure formation, and secretion of growth factors and cytokines. Key results. UG28 demonstrated the strongest pro-angiogenic activity, enhancing EC proliferation, directional migration, and pseudocapillary network formation, while CDP4 also promoted migration and early vascular organization; neither peptide showed cytotoxicity nor induced immune activation, and both were compatible with chitosan matrices. Conclusion and implications. CPNE7-derived peptides—especially UG28—are potent and safe pro-angiogenic agents with high translational potential, and their compatibility with chitosan supports their applicability in next-generation regenerative therapies targeting endothelial and microvascular repair.

## Graphical Abstract

**Figure:**
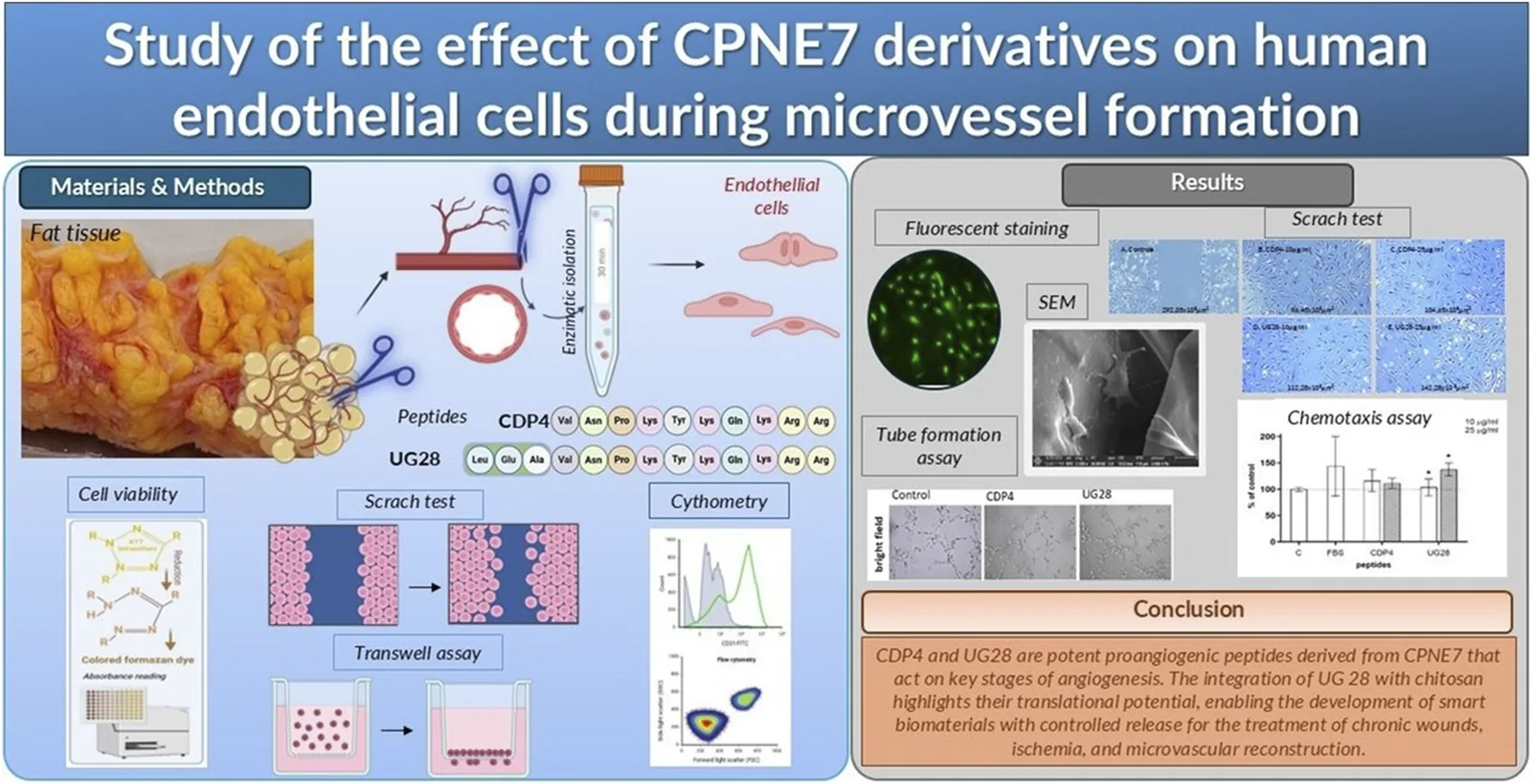

## 1 Introduction

Angiogenesis is a fundamental process required for tissue regeneration, wound healing, and the restoration of microcirculation. Impairment of endothelial function leads to chronic ischemia, delayed healing, and the progression of cardiovascular diseases, which remain among the leading causes of morbidity and mortality worldwide (Pober and Sessa, 2007; Xu et al., 2021). Despite advances in biological therapies, there is still a lack of molecules that are simultaneously effective, stable, immunologically safe, and compatible with biomaterial integration. Classical growth factors such as VEGF and FGF are limited by their short half-life, high susceptibility to degradation, and risk of adverse effects, which restricts their clinical applicability (Apte et al., 2019; Ylä-Herttuala et al., 2017; Lee et al., 2025; Berger and Hiatt, 2012).

In recent years, short bioactive peptides have gained increasing attention as an alternative to large proteins. Peptides can mimic extracellular matrix functions, modulate cell adhesion, activate surface receptors, and stimulate the secretion of growth factors (Ligorio and Mata, 2023; Wei et al., 2024; Berdiaki et al., 2024). Emerging studies indicate that pro-angiogenic peptides can effectively support vascular regeneration, with more predictable and safer activity compared to recombinant growth factors (Zhang et al., 2023; Shi et al., 2023; Zhang et al., 2024). Their small size, high stability, low immunogenicity, and suitability for precise synthesis make them one of the most promising classes of molecules in regenerative medicine (Perestenko et al., 2010).

Particular interest has been directed toward peptides derived from the Copine (CPNE) family, a group of Ca^2+^-dependent proteins involved in cellular signaling, regeneration, and differentiation (Perestenko et al., 2010; Lee et al., 2019; Khvotchev and Soloviev, 2024). Recent reports confirm that CPNE7 plays an important role in tissue repair, including neurogenesis, odontogenesis, and epithelial regeneration (Khvotchev and Soloviev, 2024; Park et al., 2019; Choung et al., 2019). However, CPNE7-derived peptides have never been investigated for their effects on human endothelial cells, representing a significant gap in the literature. No data are available regarding their influence on proliferation, migration, chemotaxis, or capillary-like structure formation—processes essential for angiogenesis.

In this study, we provide the first comprehensive evaluation of CPNE7-derived peptides—CDP4, UG28, and CPLG-UG28—on human endothelial cells. We assessed their effects on angiogenic functions, secretory profiles, and immunological safety, representing a critical step toward their potential clinical application. In our experiments, we used two peptide concentrations that had previously been verified as biologically active in an ADMSC model and consistently elicited a measurable cellular response (Tymińska et al., 2024).

In the context of future translational use, we also examined the feasibility of integrating these peptides with chitosan—a biocompatible biomaterial that recent studies highlight as a promising carrier for peptides and drugs in advanced wound dressings and tissue-engineering scaffolds (Krishna et al., 2025; Shah et al., 2025; Wiśniewska-Wrona et al., 2026). Although chitosan is not the primary focus of this work, its properties provide a practical pathway for the application of CPNE7-derived peptides in regenerative medicine.

Our findings fill an important knowledge gap by providing the first evidence of the pro-angiogenic activity of CPNE7-derived peptides in human endothelial cells and by demonstrating their potential as novel, safe, and functional molecules supporting vascular regeneration.

## 2 Materials and methods

### 2.1 Peptides

Based on the literature (Berdiaki et al., 2024), we selected the CDP4 peptide from the CPNE7 family, which has pro-regenerative properties. We added CPLG-LEA (Eckhard et al., 2016) -UG28, an amino acid sequence sensitive to the action of metalloproteinase type VII (MMP-7), which releases the 10-amino acid peptide UG28 into the environment. The peptides CDP4 (VNPKYKQKRR - ∼1315.8 Da), UG28 (LEAVNPKYKQKRR - ∼1599.9 Da) and CPLG-UG28 (CPLGLEAVNPKYKQKRR) were synthesized in solid phase using a Liberty Blue synthesizer (CEM Corporation). The synthesis was performed on ProTide resin (0.2 mmol/g) using 9-fluorenylmethoxycarbonyl (Fmoc) chemistry with side-chain-protected amino acid derivatives: Fmoc-Pro-OH, Fmoc-Glu(OtBu)-OH, Fmoc-Cys (Trt)-OH, Fmoc-Val-OH, Fmoc-Ala-OH, Fmoc-Gly-OH, Fmoc-Leu-OH, Fmoc-Lys(Boc)-OH, Fmoc-Arg(Pbf)-OH, Fmoc-Tyr(tBu)-OH, Fmoc-Gln(Trt)-OH, Fmoc-Asn (Trt)-OH. The peptides were separated from the resin within 4 h using a mixture of 88% TFA, 5% phenol, 5% deionized water, and 2% triisopropylsilane (10 mL/1 g resin in TR for 4 h). After filtration, the solutions were concentrated under vacuum, and the residue was macerated with Et_2_O. The precipitated peptide was centrifuged three times for 15 min at 4000 rpm, separating the ether phase from the crude peptide. After evaporation of Et_2_O, the peptide was dissolved in H_2_O and subjected to lyophilization.

The peptide was purified using RP-HPLC on a Kromasil C8 column (250 mm × 10 mm, 5 μm). A linear gradient from 5% B to 50% B was used over 2 h. The aqueous system (A) consisted of 0.1% (v/v) TFA in water, while the organic phase (B) consisted of 80% acetonitrile in water containing 0.1% (v/v) TFA.

Purification was monitored by UV absorption at a wavelength of 223 nm. The purity of the peptide was verified twice using LC-ESI-IT-TOF/MS (Shimadzu, Shimpol) and by RP-HPLC with a Kromasil C8 analytical column (250 mm × 4.6 mm, 5 μm), where a gradient from 5% to 100% B in A was used over 1 h, with A and B being the same as described above.

### 2.2 Functionalization of chitosan with maleimidoglycine and covalent conjugation of CPLG-UG28

Chitosan 75/500 was functionalized with maleimidoglycine to introduce maleimide groups for subsequent conjugation of a cysteine-containing peptide. The primary amino groups of chitosan were reacted with the N-hydroxysuccinimide ester of maleimidoglycine (Mal-Gly-OSu), resulting in amide-bond formation between chitosan and the maleimidoglycine linker. After the reaction, unreacted low-molecular-weight substrates were removed by filtration and washing, and the CH-Mal-Gly intermediate was dried under vacuum.

The degree of maleimidoglycine substitution was determined after acid hydrolysis of CH-Mal-Gly by hydrophilic interaction liquid chromatography (HILIC). Analyses were performed using a Nexera chromatographic system equipped with ELSD and PDA detectors and a bioZen Glycan column. The mobile phases consisted of ammonium formate/formic acid buffer in acetonitrile-water mixtures. The flow rate was 0.25 mL min-1 and the total analysis time was 45 min. Norleucine was used as the internal standard, whereas glycine released during hydrolysis was used as the analytical marker of maleimidoglycine incorporated into chitosan. Quantification of the glycine signal relative to the norleucine internal standard indicated a maleimidoglycine substitution degree of approximately 10%.

CPLG-UG28 (CPLGLEAVNPKYKQKRR), containing an N-terminal cysteine residue, was subsequently conjugated to CH-Mal-Gly. The peptide was added in excess relative to the estimated amount of maleimide groups to favor reaction of the accessible maleimide functionalities. An aqueous mixture of CH-Mal-Gly and CPLG-UG28 was incubated for 24 h at room temperature, at pH 8.2, under an inert atmosphere. Under these conditions, the thiol group of the N-terminal cysteine undergoes Michael-type addition to the maleimide group, yielding a covalent succinimidyl thioether linkage between CPLG-UG28 and the chitosan-maleimidoglycine backbone. The product was collected using a Gooch G3 filter, washed to remove excess non-bound peptide, and dried under vacuum for 1 h. The degree of chitosan substitution with maleimidoglycine was approximately 10%; the subsequent CPLG-UG28 coupling efficiency was not quantified independently.

The approximately 10% value reported for this preparation refers exclusively to the experimentally determined degree of chitosan substitution with maleimidoglycine. The coupling efficiency and final CPLG-UG28 loading were not independently quantified. Because the peptide was used in excess and the non-bound peptide was removed during purification, the available maleimide groups were assumed to be substantially consumed; accordingly, the maleimidoglycine substitution degree was treated as the nominal upper limit of peptide functionalization rather than as a directly measured peptide-loading value.

### 2.3 Chitosan hydrogel membrane

A chitosan solution with a peptide concentration of 10 μg/mL was obtained by mixing two types of chitosan, one with the peptide attached and the other without, in a mass ratio of 69:1. The chitosan solution in C_2_H_4_O_3_ was prepared according to the method developed by Gorczyca et al. (2014) and the subsequent steps were carried out as described in the publication by Tymińska et al. (2024).

### 2.4 ECs isolation, cell culture and passages

The research material consisted of human adipose tissue, which was medical waste obtained during planned surgical procedures at the Plastic Surgery Clinic, for which approval was obtained from the Independent Bioethics Committee for Scientific Research at the Medical University of Gdańsk (approval no. NKBBN/672/2019, NKBBN/672-65/2023). The material was collected from the abdominal area of 10 healthy individuals aged 22 to 57 with a BMI <30. Patients who had undergone chemotherapy/radiotherapy or individuals with a BMI>30 were excluded from the study.

The tissue collected from patients was mechanically cleaned of perivascular adipose tissue using a scalpel and washed in PBS with 2% antibiotic to remove erythrocytes from the lumen of the vessels. In the next stage, the material was mechanically fragmented into 2–3 mm pieces and rinsed with PBS solution. The fragments were placed in a solution of dispase enzyme at a concentration of 0.15 U/mL and collagenase IA at a concentration of 0.15 U/mL, and 10 mL of ECGM MV endothelial cell medium. The fragments were incubated at 37 °C for 1 h, then neutralized with PBS containing antibiotic. They were then neutralized with medium and passed through a 100 µm pore diameter filter. After centrifugation, the cell pellet was resuspended in medium and seeded onto 3 cm diameter plates coated with type IV collagen. After 24 h, the medium was replaced to remove non-adherent cells. The cultures on the plates were maintained for approximately 7–10 days until the cells reached 80% confluence. After this time, the cells were lysed with trypsin, washed and transferred to culture bottles. The medium was replaced every 2–3 days, and cells in the second passage (p2) were collected for experiments.

### 2.5 Confirmation of endothelial phenotype

#### 2.5.1 Flow cytometry

Positive surface markers: CD31 (PECAM1), LDLR, CD133; eNOS, CD146 (S-endo-1), CD309 (VEGFR2) and negative marker: CD14 were used to assess the phenotype of endothelial cells (Table S1). Flow cytometry was used Cytoflex - Beckman Coulter. For this purpose, cells were trypsinised, neutralized, centrifuged and counted. 1 × 10^5^ cells were divided by adding 5 µL of each antibody and incubated for 30 min. The control sample consisted of cells not labelled with antibodies. The cells were then washed and examined cytometrically.

#### 2.5.2 Functional potential—Capillary formation test

For the capillary formation assay, the so-called reconstituted basement membrane - Matrigel was used (Corning Life Sciences). The matrigel, which is commercially available, was originally obtained from the basement membranes of mouse EHS sarcoma (Engelbreth-Holm-Swarma).

Matrigel (approximately 100 µL) was poured into 48-well plates on ice. Thirty minutes before cell seeding, the gel plate was placed in an incubator with CO_2_, 5%, 37 °C to polarize its surface. Endothelial cells at passage two, were seeded at 2 × 10^4^ per well in 300 µL of dedicated ECGM MV medium (PromoCell, Germany), were cultured for 4 h and photographed every 1 h under an inverted light microscope.

### 2.6 Proliferation test (XTT)

The XTT assay (Cell Proliferation Kit II; Sigma Aldrich) measured the viability of peptide-stimulated ESCs. For this, 5 × 10^3^ cells were seeded into a well (96-well plates) in ECGM MV medium with FBS and antibiotics. After 24 h, the medium was exchanged for a serum-poor medium (2%) with selected peptide concentrations of 10 μg/mL (CDP4 - 7.6 μM, UG28–6.25 µM) and 25 μg/mL (CDP4 – 19,0 μM, UG28–15,6 µM). Cells were incubated with the peptide for 48 and 72 h. After this time, XTT reagent was added, absorbance was read at OD 450 nm, after 4 h of incubation, at 37 °C and in the presence of 5% CO_2_. Controls were cells not treated with peptides with 2% FBS (C - control; set as 100%) and a positive control with 5% FBS.

Fluorescence staining was performed using a 1 µL solution of Calcein-AM and propidium iodide mixed with PBS at a ratio of 1 µL per 1 mL. Cells stained on chitosan membranes were observed under a fluorescence microscope (OLYMPUS) capable of detecting green and red fluorescence.

### 2.7 Cytotoxicity assay (LDH)

The cytotoxicity of the compounds was tested by an LDH assay measuring lactate dehydrogenase (LDH) activity in cell culture supernatants. ECs were seeded into 96-well plates. Cells cultured 48 h in FBS-poor medium were then stimulated with peptides. After the incubation time, supernatants were harvested and analyzed by LDH assay, according to the manufacturer’s instructions (Takara, Japan). Cell death was compared with the peptide-untreated control and the positive control (with maximum LDH release; 100% cytotoxicity) induced with Triton X-100 detergent. Absorbance values were read at 450 nm (absorbance reader SYNERGY, BioTek).

### 2.8 Migration test (scratch test)

Cell migration was measured using the scratch assay. Cells were seeded into culture dishes with a silicon separator (from Ibidi, Germany) at 2 × 10^4^ cells each for 24 h in EC MVC medium supplemented with 5% FBS. After this time, the separator was removed and cell proliferation was blocked by adding mitomycin C (5 μg/mL) for 2 h, and the medium was changed to 2% FBS with peptides at two concentrations (10 and 25 μg/mL). After a further 24 h, the migration of ECS to the inside of the scratch was analyzed. Cells were fixed with 3.7% PFA and stained with 0.05% crystal violet, and the effect of migration was assessed by measuring the unoccupied area in the scratch.

### 2.9 Chemotaxis assay

Cells in passage two were cultured under FBS-poor conditions: ECGM with 2% FBS, for 24 h at 37 °C and 5% CO_2_. After 24 h, cells were detached and placed on the inner membrane of the insert (pore size 8.0 μm). The membrane of the insert divided the well into an upper part, where 1 × 10^5^ cells were seeded in 200 µL of medium, and a lower part, where 600 µL of FBS-poor medium with a specific concentration of peptide was present. The controls consisted of cells with 2% and 5% FBS without peptide. Plates were incubated for 24 h, then cells were labelled for 45 min with 8 μM calcein solution in the medium. Cells that had migrated to the outer membrane of the insert were detached with trypsin. The fluorescence of the cells was then read at an excitation wavelength of 485 nm and emission wavelength of 520 nm (absorbance reader SYNERGY, BioTek). Absorbance values were converted to a percentage relative to the control sample.

### 2.10 Luminex®MAP® method-quantification of cytokines and growth factors

The Luminex®MAP® method was used to assess the paracrine capacity of EC cells, which enables the detection of multiple proteins present in the supernatant in a single biological test. The concentrations (pg/mL) of the following cytokines were examined in the supernatant of cultured AD-SC and EC cells: EGF, G-CSF, leptin, FGF-1, IL-8, HGF, HB-EGF, VEGF-C, VEGF-D, FGF-2, VEGF-A. The MILLIPLEX®MAP® Human Angiogenesis/Growth Factor Magnetic Bead Panel 1 (HAGP1MAG-12K; Merck Millipore, USA) was used for the experiments. Supernatant samples from four patients were thawed on ice. The assays were performed according to the manufacturer’s instructions. Supernatant samples were incubated with colour-coded magnetic beads coated with cytokine-specific capture antibodies. A mixture of biotinylated detection antibodies was then added to bind to the capture antibodies. In the next step, streptavidin conjugated with phycoerythrin was added. The samples prepared in this way were read using a Luminex MAGPIX® analyzer. xPONENT 4.2 software was used for data analysis.

### 2.11 Immunogenicity

The immunogenic potential of the peptides was tested on human peripheral blood cells (PBMC), isolated from “buffy coats” using Ficoll. PBMCs were seeded at 1 × 10^6^/ml of RPMI 1640 medium (P/S, 10% FBS) per well. After 1 day, they were stimulated with peptide at a 10 μg/mL concentration for 24 h. The negative control was PBMC without compound and the positive control was LPS-stimulated cells; 1 μg/mL. Cells were then washed and labelled with anti-CD3, CD4, CD8, CD25, CD69, CD71 and HLA-DR (concentration 1:100). After 30 min incubation at room temperature in the dark, they were analyzed by cytometric method - Cytoflex (Beckman Coulter).

### 2.12 Basophil activation test (BAT)

The basophil activation test (BAT) was performed using an allergy cell analysis kit (Beckman Coulter, USA). Peripheral blood was collected from healthy patients into EDTA tubes. One hundred μl of blood was treated with 20 μL of peptides (10 and 25 μg/mL) as well as filtrate taken from above the lyophilizate and chitosan gel. CD3, CD203c and CRTH2 were then added (concentration 1:100). Samples were incubated for 15 min at 37 °C in a water bath, erythrocytes were lysed, washed and analyzed cytometrically. A negative control and positive controls were used. Cut-off values for the allergen analyzed were set as 5% of the negative control sample after stimulation.

### 2.13 Assessment of the functional differentiation of ECs on matrigel with CDP4 and UG28 peptides

Matrigel was dispensed into 48-well plates and incubated for 30 min at 37 °C. After polymerization, endothelial cells at passage two were seeded onto the gel surface at a density of 5 × 10^3^. CDP4 and UG28 peptides were added to the wells at a concentration of 10 μg/mL. The experiment was conducted for 1–4 h, after which the cells were stained with calcein to assess cell viability and capillary formation.

### 2.14 Assessment of the functional differentiation of ECs on a chitosan lyophilizate conjugated with UG28

A chitosan lyophilizate conjugated with UG28 at a concentration of 10 μg/mL was placed in the wells of a 48-well plate. Endothelial cells at passage p2 were seeded onto the surface of the lyophilizate at a density of 5 × 10^3^. The experiment was conducted for 72 h, after which the cells were treated with a glutaraldehyde solution as described below.

### 2.15 SEM (scanning electron microscopy)

Samples were initially fixed in a 2.5% glutaraldehyde solution (Agarscientific, UK) in phosphate-buffered saline for a duration of 4 hours, followed by overnight post-fixation in 1% osmium tetroxide (Agarscientific, UK). Subsequently, the samples underwent a gradual dehydration process in ethanol and were subsequently dried using 1.1.1.3.3.3-hexamethyldisilazane (HMDS) (Sigma-Aldrich, USA). After an air-drying period of 24 h, the samples were coated with gold using a sputter coater and subsequently examined under a Prisma E scanning electron microscope (Thermo Fisher, USA).

### 2.16 Statistical analysis

The data represents the mean ± SEM from four independent experiments (with four replicates in each). The (normal) distribution of the data was tested using the Shapiro–Wilk test; if the *P*-value is significantly less than 0.05, the null hypothesis of normality is rejected.

Comparisons between groups were performed using the Mann–Whitney *U* test. Dunn’s *post hoc* analysis—comparisons using the Mann–Whitney *U* test corrected by the Holm method—confirmed significant differences between the experimental groups. Statistical analysis was performed Statistica 13.3, and for the graphical presentation of the results, the program GraphPad Prism 5.0.

## 3 Results

### 3.1 Confirmation of ESCs phenotype

#### 3.1.1 Cytometric analysis and capillary formation test

**FIGURE 1:**
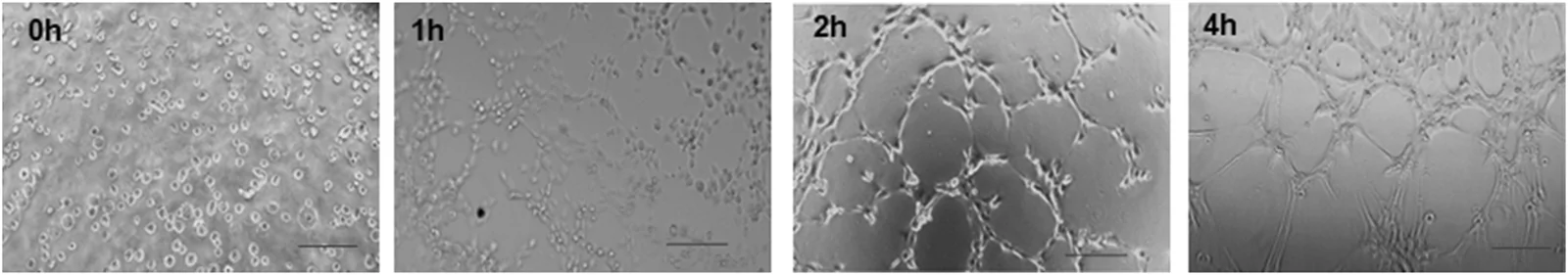
Evaluation of cell phenotype in the pseudocapillary formation assay at 0, 1, 2, 4 h after seeding on Matrigel. Magnification ×100, Bar 200 µm.

The capillary formation assay also confirmed the extensive formation of loops, nodes and branching cellular connections forming capillary-like networks (Figure 1). The isolated cells showed high expression of endothelial-specific markers with a low percentage of negative markers (Table 1; Supplementary Figure S1), confirming their endothelial phenotype.

**TABLE 1:** Confirmation of the endothelial phenotype of cells isolated from adipose tissue shows the specific positive and negative markers for ECs. Table S1. Fluorescently labeled antibodies and dyes (references).

Endothelial cell markers	​	​	​	​	​	​
Positive	CD31	CD133	CD146	CD309	eNOS	LDLR
​	90.81%	98.13%	98.17%	99.17%	99.99%	99.34%
Negative	CD14	​	​	​	​	​
​	1.43%	​	​	​	​	​

### 3.2 Proliferation assay

**FIGURE 2:**
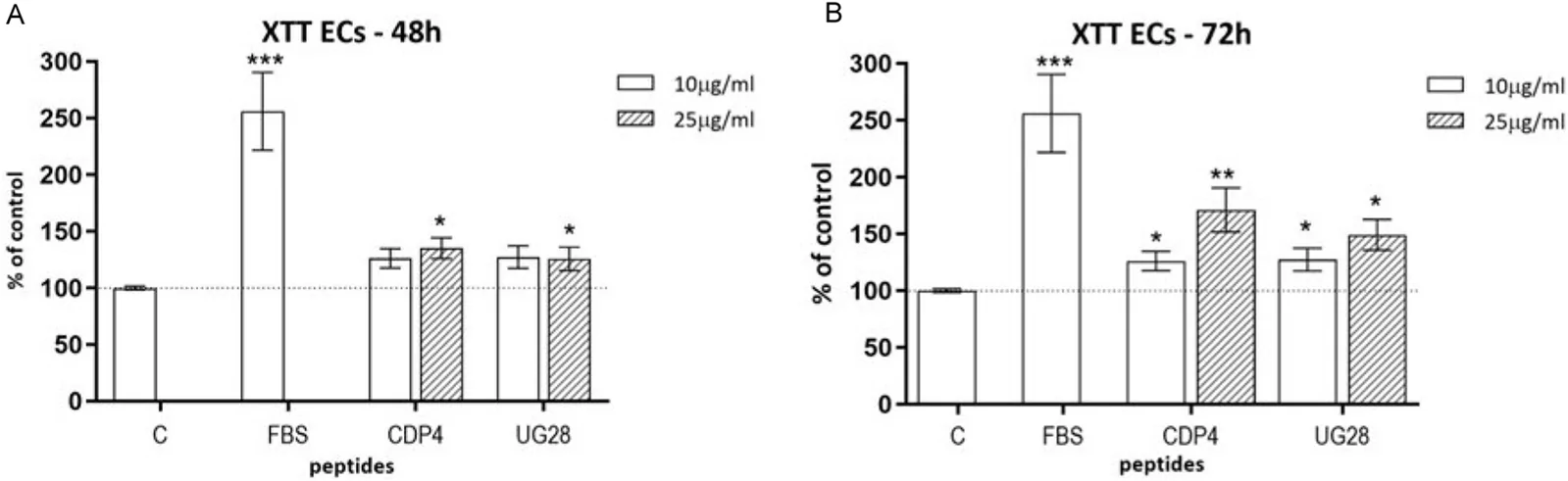
Evaluation of cell proliferation of ECs stimulated with CDP peptide derivatives after 48 h **(A)** and 72 h **(B)** of incubation. Graph shows mean ± SEM from four independent experiments (4 replicates in each, n = 16). Significantly statistical differences compared to control without peptides (cells cultured with FBS-poor 2% medium). Dunn’s *post hoc* analysis—comparisons using the Mann–Whitney *U* test corrected by the Holm method ***P < 0.0001—highly significant, **P < 0.001—significant, *P < 0.05—marginally significant, Comparison between groups, Supplementary Figure S7.

CPNE7 peptides at concentrations of 10 and 25 μg/mL had a marked effect on endothelial cell proliferation, with the strongest response observed for CDP4 (25 μg/mL), which increased cell numbers in a concentration-dependent manner. This indicates that CDP4 activates cell cycle-related pathways and promotes endothelial cell expansion, which is a key step in angiogenesis. UG28 also increased proliferation, albeit to a lesser extent, suggesting a more limited but still significant effect on regenerative processes. These findings confirm that CPNE7-derived peptides can function as proangiogenic factors by stimulating cell growth (Figure 2).

None of the peptides exhibited cytotoxicity at the tested concentrations, confirming their biological safety. The cells maintained normal morphology and viability, which is essential for potential clinical applications. This finding distinguishes CPNE7-derived peptides from many growth factors that can display adverse effects at higher doses (Supplementary Figure S2).

### 3.3 Migration test (scratch test)

**FIGURE 3:**
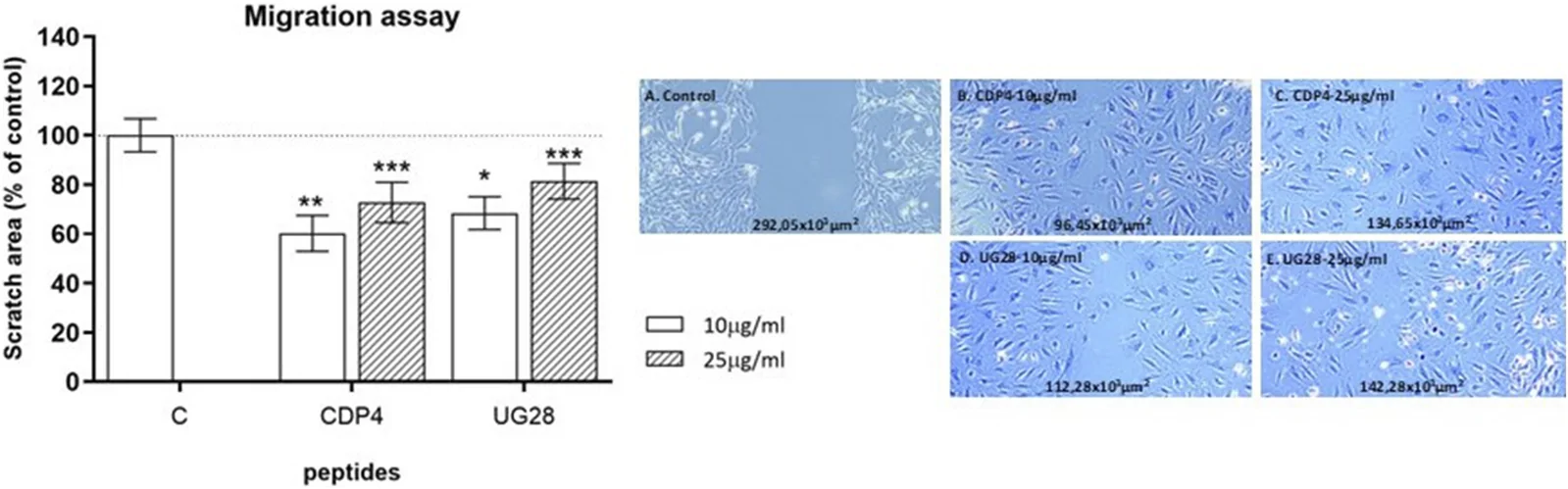
The effect of CDP4 peptides, UG28, in two concentrations, on endothelial cell migration after 24 h of incubation. The graph shows the mean ± SEM from four independent experiments (each with four replicates). Statistically significant differences - scratch area hypertrophy reduces the bar value resulting from endothelial cell migration compared to the control (C). Dunn’s *post hoc* analysis—comparisons using the Mann–Whitney *U* test corrected by the Holm method ***P < 0.0001—highly significant, **P < 0.001—significant, *P < 0.05—marginally significant, Comparison between groups, Supplementary Figure S8.

Peptides increased endothelial cell migration, but CDP4 showed the strongest and most dynamic effect in the scratch test. The faster closure of the gap in the monolayer indicates that CDP4 activates cytoskeletal reorganization and cell-adhesion mechanisms, which are essential for new vessel formation. UG28 also improved migration, but its effect was more moderate and primarily evident at early stages. This suggests that UG28 may play a supportive role, whereas CDP4 serves as the main driver of endothelial cell migration (Figure 3).

### 3.4 Chemotaxis assay

Chemotaxis demonstrates the ability of cells to move purposefully in response to chemical signals in their environment. It is essential for life processes and ensures regeneration and wound healing.

**FIGURE 4:**
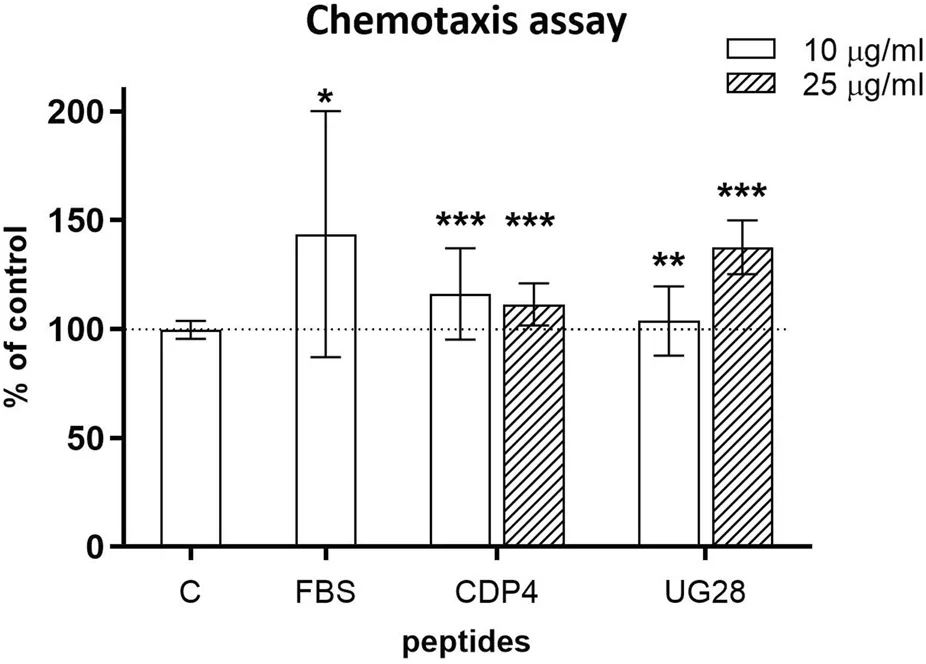
Effect of CDP4 and UG28 peptides at two concentrations on chemotaxis of ECs after 24 h incubation. The graph shows the mean ± SEM from four independent experiments (each in four replicates). Significantly statistical differences compared to control without peptides (cells cultured with FBS-poor 2% medium). Dunn’s *post hoc* analysis—comparisons using the Mann–Whitney U test corrected by the Holm method ***P < 0.0001—highly significant, **P < 0.001—significant, *P < 0.05—marginally significant, Comparison between groups, Supplementary Figure S9.

In the chemotaxis assay, UG28 induced the strongest directional response, indicating that endothelial cells actively and coordinately respond to its gradient. This is a characteristic feature of peptides with high angiogenic potential. CDP4 also enhanced chemotaxis, but to a lesser extent, suggesting that its effect may be more related to non-directional rather than directional migration. These findings indicate that UG28 may function as a chemoattractant signal guiding cells toward the site of regeneration (Figure 4).

### 3.5 Functional potential of CDP4 and UG28 peptides in capillary formation on matrigel

**FIGURE 5:**
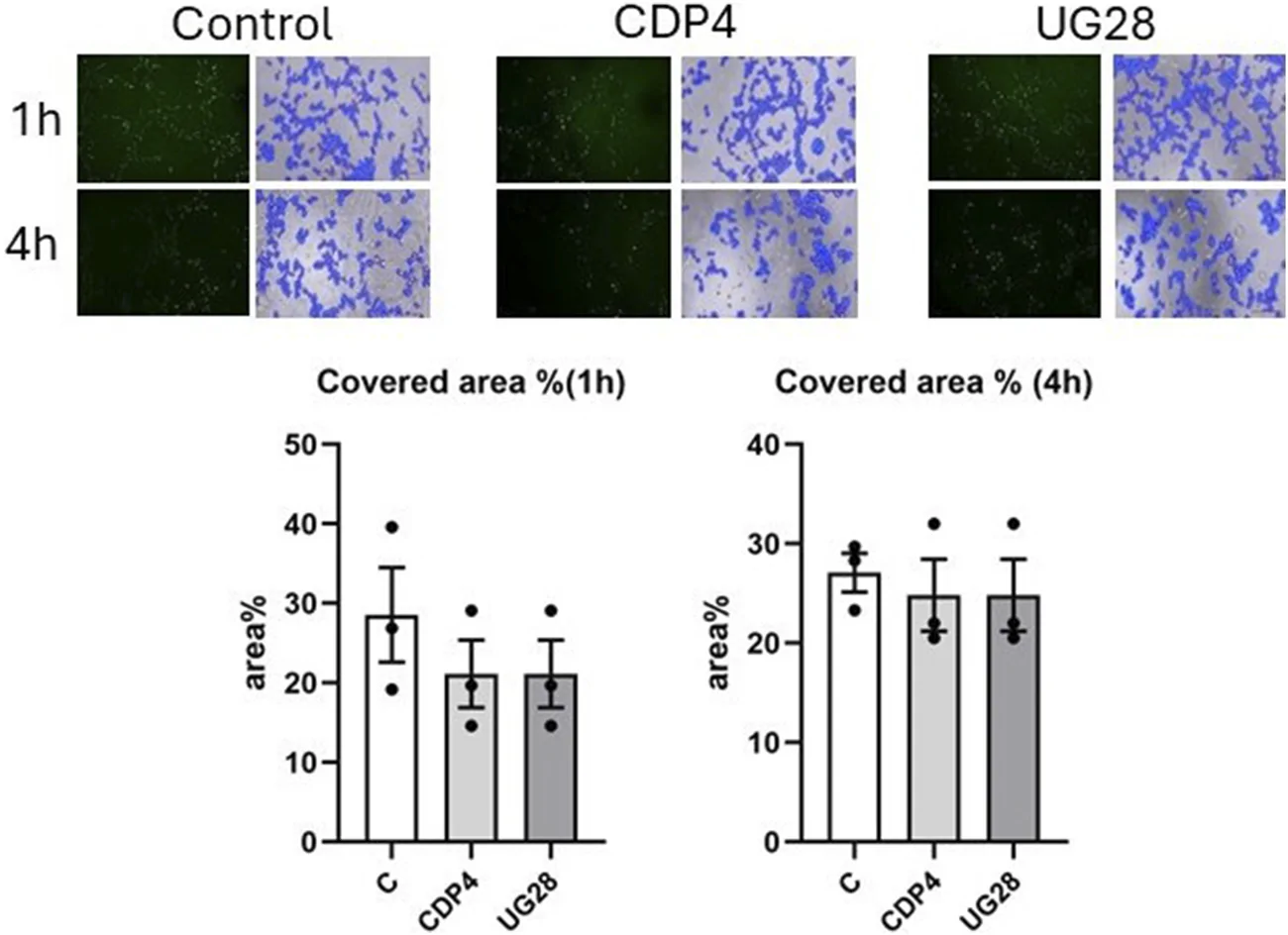
Tests for the formation of microcapillaries on a Matrigel substrate. Representative images of cells covering the substrate after 1 and 4 h under control conditions and after stimulation withCDP4 and UG28 peptides. The images were obtained using a fluorescence microscope at ×100 magnification, and the percentage of gel coverage by cells was determined using the Wimasis.com image analysis software. The graphs show the percentage of Matrigel surface covered by endothelial cells after stimulation with peptides at a concentration of 10 μg/mL (n = 3).

**FIGURE 6:**
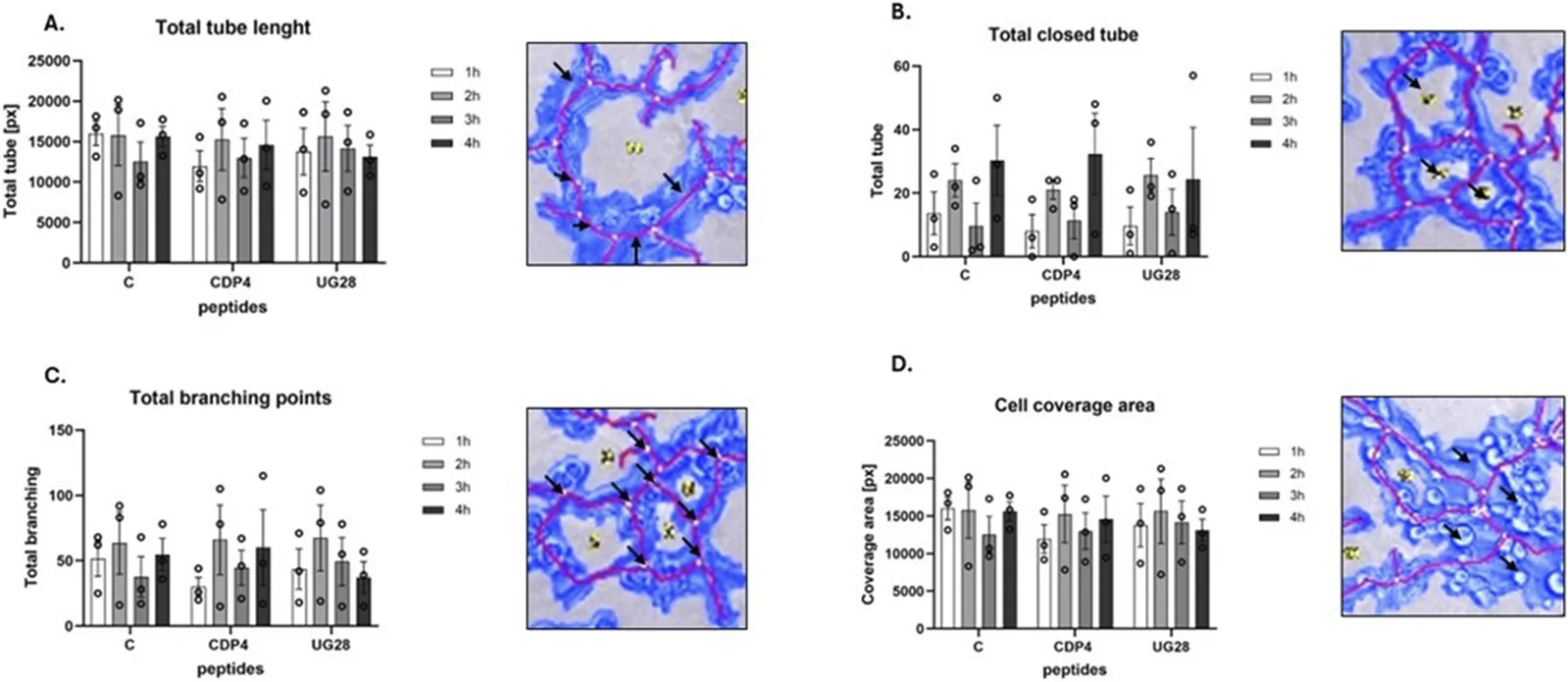
The graphs show the characteristics of pseudocapillaries in terms of vessel network growth, branching points, and wall length **(A)** shows the total tube length **(B)** shows the number of closed “meshes,” **(C)** shows the number of branches formed by ECs, and **(D)** shows the total area of Matrigel covered by ECs. The arrows indicate individual elements of the capillary networks.

**FIGURE 7:**
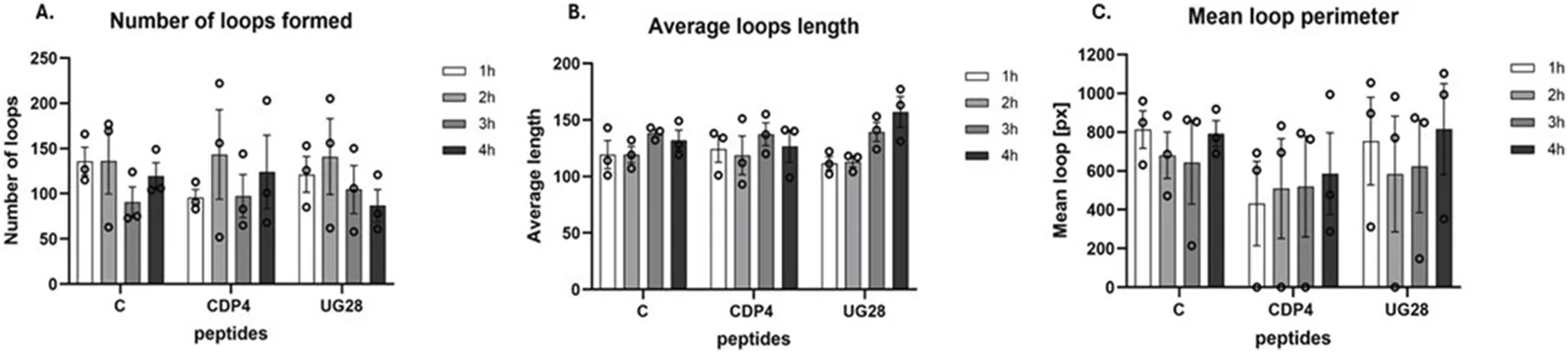
The graphs show the characteristics of the number of loops created **(A)**, the average length of the loops **(B)**, and the mean loop perimeter **(C)**. The study was performed on three patients (n = 3), and the Mann–Whitney *U* test did not show statistical significance.

UG28 significantly increased the number, length, and stability of capillary-like structures, indicating its ability to activate the full angiogenic program (Figure 5). Endothelial cells treated with UG28 formed more branched and stable networks, reflecting the activation of both early and late stages of vessel formation. CDP4 also induced capillary formation, but the resulting structures were shorter and less developed. This indicates that while CDP4 supports angiogenesis, its effect is not as pronounced as that of UG28 (Figures 6 and 7). Overall, the results clearly show that UG28 is the most promising peptide in the context of vascular regeneration.

#### 3.5.1 Capillary characteristics

ECs were examined for the characteristics of developing capillaries after stimulation with peptides compared to controls on Matrigel.

The studies showed the highest total vessel length after 2 h of culture with each peptide, compared to the first hour after cell seeding. The following hours brought a decrease in wall length after UG28 treatment. In contrast, the addition of the CDP4 peptide resulted in effects very similar to the controls (Figure 6A). The highest number of completely closed tubes was observed after 4 h of cell culture. The results fluctuated around the control values, with the highest value observed after CDP4 stimulation in the fourth hour of the study, but without statistically significant differences (Figure 6B). The number of pseudopodia branches was highest after 2 h of cell culture. In the third hour, the number of branching points decreased for all conditions and then increased for the control and CDP4 peptide (Figure 6C). The area occupied by the cells was equal to the total tube length (Figure 6D).

#### 3.5.2 Loop characteristics

The number of loops forming by cells over a period of 1–4 h was measured. Results showed the highest effect after 2 h of cultivation with the CDP4 peptide by as much as 30% and for UG28 by 20%. Cells in the control group without stimulation did not exceed the number of loops forming in each subsequent hour of the experiment (Figure 7A). The average loop length was highest after UG28 stimulation after 4 h of the experiment, and the circumference was largest in comparison with C and CDP4 after 1 h of the experiment (Figure 7B,C).

### 3.6 Assessment of functional differentiation of ECs on chitosan lyophilizate with a covalently conjugated peptide UG28

**FIGURE 8:**
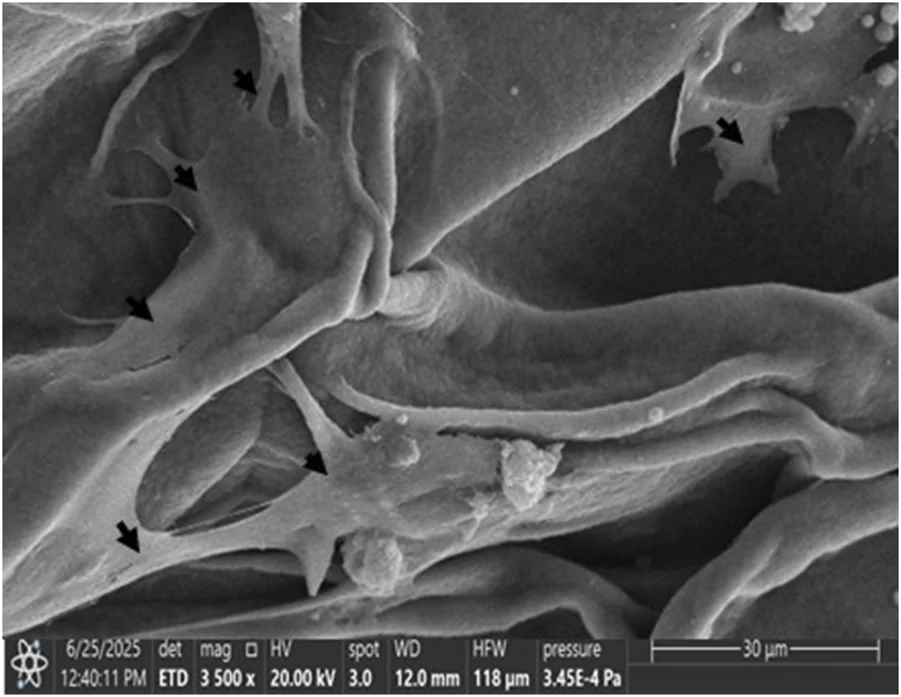
The image, taken using scanning electron microscopy, shows a chitosan lyophilizate with the attached UG28 peptide and endothelial cells indicated by arrows.

A lyophilized chitosan-CPLG-UG28 construct was obtained using the thiol-maleimide conjugation protocol. Long-term conjugate stability and peptide release were not evaluated in the present study (Figure 8). Although their activity within the biomaterial was not evaluated, these findings suggest that CPNE7-derived peptides may be suitable for future use in smart wound dressings or tissue scaffolds. This represents an important step toward translational applications.

## 4 Discussion

In this study, we demonstrate that CPNE7 family peptides exert a dose-dependent effect on endothelial cells, influencing key processes involved in vascular reconstruction. Building on our earlier findings in adipose-derived stromal cells, these results extend the biological relevance of CPNE7 peptides beyond osteogenic and chondrogenic contexts and suggest previously unrecognized role in endothelial regeneration. Given the central importance of angiogenesis for tissue repair and regenerative therapies, these findings provide new insight into the therapeutic potential of short bioactive peptides for vascular modulation.

Based on our previous studies on ADSC cells, we selected the two most promising peptide concentrations to precisely evaluate their biological effects on endothelial cells and to determine the dose–response relationship in key angiogenic processes. This approach follows current preclinical standards for short bioactive peptides, which often display biphasic activity, as highlighted in recent reviews on angiogenesis regulation and therapeutic modulation (Choi, 2025; Gan et al., 2022; Pham et al., 2024). The lower concentration (10 μg/mL) allowed us to assess whether the peptides exhibit activity at minimal cellular exposure, which is important for safety and potential clinical applications (Figure 2A,B). A similar strategy has been applied in recent studies on pro-angiogenic peptides such as peptide Lv, which also shows a clear dose-dependent response in endothelial cells (Pham et al., 2024). The higher concentration (25 μg/mL) enabled us to evaluate whether increasing the dose enhances the pro-angiogenic effect or leads to saturation or attenuation—phenomena typical for many short regulatory peptides. Using two concentrations also allowed us to assess biological safety, as potential cytotoxic, immunological, or migration-inhibiting effects often emerge only at higher doses.

This strategy is widely used in research on CPNE7-derived peptides and their integration with biomaterials, as confirmed by recent work on CPNE7 derivatives and chitosan-based substrates (Tymińska et al., 2024). This approach has made it possible to determine the angiogenic potential of CDP4 and UG28 and their suitability for future biomaterial-based applications, in line with current trends in regenerative therapies using short bioactive peptides (Pham et al., 2024; Hao et al., 2024; Vishwanath et al., 2025). Both peptides have a positive C-terminal KQKRR sequence, which confers CPP-like properties and enables cellular penetration. An additional LEA segment in UG28 stabilizes the structure and enhances membrane interactions, thereby increasing its activity. CDP4 and UG28 are functional CPNE7 microdomains that retain the ability to interact with the membrane, modulate the cytoskeleton, and activate signaling.

The chitosan-CPLG-UG28 construct used in this study was prepared by a two-step covalent functionalization strategy rather than by physical loading, adsorption, ionic complexation, or emulsion electrospinning. Maleimidoglycine was first coupled to the primary amino groups of chitosan through an activated-ester reaction. The approximately 10% substitution degree determined by HILIC therefore describes the density of maleimidoglycine groups introduced into the chitosan backbone (Supplementary Figure S10). In the subsequent step, the N-terminal cysteine of CPLG-UG28 was allowed to react with the maleimide functionalities through thiol-maleimide Michael addition. This site-directed reaction was selected to favor covalent immobilization of the peptide precursor while preserving the remaining peptide sequence.

The distinction between covalent and non-covalent incorporation is relevant because chitosan can also associate with peptides through electrostatic interactions, hydrogen bonding, and hydrophobic interactions. In the present procedure, the use of a cysteine-bearing peptide, maleimide-functionalized chitosan, reaction conditions favorable for thiol-maleimide addition, and removal of excess peptide support the intended covalent-conjugation pathway. Nevertheless, the final peptide loading was not measured directly. The peptide was added in excess, and substantial consumption of the accessible maleimide groups was assumed; thus, the measured approximately 10% maleimidoglycine substitution represents a nominal upper limit for peptide functionalization rather than direct proof of an equivalent peptide substitution degree.

The present results should therefore be interpreted as demonstrating the feasibility of preparing a lyophilized chitosan-CPLG-UG28 construct by a defined covalent-conjugation protocol. SEM confirmed the morphology of the construct and the presence of endothelial cells on its surface under the tested culture conditions, but this analysis cannot verify linkage identity, coupling efficiency, or bond stability. Moreover, no dedicated release or long-term stability study of the chitosan-CPLG-UG28 construct was performed in PBS, cell-culture medium, serum-containing medium, or in the presence of MMP-7. Consequently, covalent attachment should not be equated with experimentally demonstrated long-term stability or controlled release.

The CPLG-UG28 design contains an enzyme-responsive segment intended to permit proteolytic liberation of the UG28-related bioactive sequence. Establishing this function will require quantitative determination of the final peptide loading, analysis of unreacted maleimide and/or thiol groups, monitoring of peptide or peptide fragments in wash and incubation media, and time-resolved release studies under physiologically relevant conditions, including MMP-7-containing media. These experiments will be necessary to distinguish true enzyme-triggered release from desorption of residual non-covalently associated peptide and to define the long-term stability and delivery profile of the construct.

CDP4 significantly increased endothelial cell proliferation, while UG28 induced a weaker but still clearly proangiogenic effect (Figure 2A,B). This activity profile is consistent with the current understanding of angiogenesis as a multi-step process in which endothelial proliferation is a critical early phase of blood vessel regeneration in wound healing, ischemia, and cardiovascular disease (Shi et al., 2023; Choi, 2025; Kalies et al., 2025). At the same time, our results show that the response of cells to peptides is dose-dependent: too low doses are ineffective, while too high concentrations can lead to a plateau or even inhibitory effects, a phenomenon widely described for short regulatory peptides (Hao et al., 2024; Ciulla et al., 2023; Tetè et al., 2025). In migration assays, CDP4 accelerated scratch closure in endothelial monolayers more strongly than UG28, suggesting a more potent activation of endothelial motility mechanisms involved in neo vessel formation (Figure 3). Recent literature emphasizes that effective pro-angiogenic therapies must support both proliferation and migration, as increasing cell numbers without enabling their directed movement does not result in functional neovascularization (Lee et al., 2022; Luo et al., 2023; Alders et al., 2025). CDP4 therefore appears to act as a modulator of early angiogenic stages, whereas UG28 may function as a supportive but less potent factor.

In the chemotaxis assay, UG28 induced strong, gradient-dependent directional migration, while CDP4 triggered a more moderate response (Figure 4). This distinction is highly relevant, as in ischemic conditions the key requirement is not only enhanced motility but also precise guidance of endothelial cells toward the damaged region. Contemporary reviews highlight that chemotactic gradients are central to the spatial organization of newly forming vessels (Ferre-Torres et al., 2023; Stokes et al., 1990; Pellet-Many and Fiedler, 2015; Minerva et al., 2022). Our findings suggest that UG28 may act as such a directional cue, analogous to classical chemokines or growth factors but potentially with fewer adverse effects than VEGF/FGF-based therapies, which are known to induce unstable, leaky, and poorly organized vessels (Venkatraman et al., 2019; Pérez-Gutiérrez and Ferrara, 2023). In contrast, the networks formed under UG28 stimulation were more ordered and stable, indicating that CPNE7-derived peptides may promote more mature and functional angiogenesis than VEGF monotherapy.

Further evidence of the pro-angiogenic activity of CPNE7 peptides comes from the tube-formation assay. UG28 significantly increased the number, length, and branching of capillary-like structures, resulting in a dense and stable network. CDP4 also supported tube formation, but the resulting structures were shorter, less branched, and less stable (Figures 5–7). These differences are consistent with findings from studies in which bioactive peptides or ECM-mimetic proteoglycan analogs incorporated into tissue scaffolds enhanced neovascularization and improved wound healing, including in diabetic models (Demenj et al., 2025; He et al., 2022; He et al., 2021). Our results show that UG28 can induce a similar pro-angiogenic phenotype even without a scaffold, acting directly on endothelial cells.

UG28 also increased the secretion of key pro-angiogenic factors such as VEGF-D and EGF, HGF (Supplementary Figure S3). This mechanism aligns with the emerging concept of “indirect” angiogenesis modulation, where instead of administering exogenous VEGF, endothelial cells are stimulated to produce their own regulated pro-angiogenic signals (Flournoy et al., 2022; Lungu et al., 2025). Reviews on neovascularization in chronic wounds and ischemia emphasize that therapies supporting endogenous angiogenic programs may be safer and lead to more physiological vessel remodeling (Shi et al., 2023; Huerta et al., 2023). Our findings suggest that UG28 effectively “switches” endothelial cells into a pro-angiogenic mode by enhancing their secretory response.

A favorable result of our studies is the absence of cytotoxicity (Supplementary Figure S2) and detectable immunogenicity for both UG28 and CDP4 under the tested conditions (Supplementary Figures S4, S5). These data indicate that CPNE7-derived peptides may represent a safe alternative with targeted endothelial activity and minimal inflammatory activation.

The ability to integrate the peptides with chitosan and form stable lyophilized constructs aligns with current trends in bioactive scaffold development, where mechanical and biochemical cues are combined within a single system. Studies on ECM-mimetic matrices and progenitor-cell-loaded scaffolds show that such approaches significantly enhance neovascularization, particularly in challenging environments such as diabetic wounds (Zhang et al., 2024; He et al., 2022; Mangani et al., 2025). Our results suggest that UG28 (and, to a lesser extent, CDP4) may act as key bioactive components in such systems, with the construct serving as a potential platform requiring quantitative validation in terms of loading, release and stability (Figure 8).

Although we have not yet evaluated functional angiogenesis in chitosan–peptide systems, the successful integration alone is consistent with the concept of next-generation “smart” biomaterials that deliver pro-angiogenic signals locally and in a controlled manner.

From the perspective of current knowledge about angiogenesis and its regulation, our research provides new information on the action of short peptides from the CPNE7 family, which may support the process of capillary formation, their growth and participation in the regeneration of difficult-to-heal wounds. Recent reviews emphasize that angiogenesis is regulated by complex networks of pro- and anti-angiogenic signals involving growth factors, inflammatory cells, and interactions with the ECM (Choi, 2025). At the same time, the limitations of therapies based on single large molecules such as VEGF are increasingly recognized, including short half-life, poor spatiotemporal control, and the risk of adverse effects (Santorsola et al., 2024; Wang et al., 2024).

CPNE7-derived peptides—in particular UG28 — fit into the concept of a new generation of angiogenesis modulators. They are characterized by their small size, high stability, selective activation of endothelial cells and ability to enhance natural proangiogenic programmers. Furthermore, they are compatible with the integration of biomaterials designed for local delivery of molecules (Supplementary Figure S6). When combined with biomaterials such as chitosan, they can form systems that simultaneously provide structural support and precise modulation of angiogenesis processes.

This study provides a preliminary assessment of the pro-vasculogenic potential of the CDP4 and UG28 peptides. The results obtained, which include an analysis of endothelial cell migration, the formation of capillary-like structures and VEGF secretion, indicate their ability to stimulate processes associated with vasculogenesis and angiogenesis. It should be emphasized, however, that the *in vitro* models used do not fully reflect the complexity of the tissue microenvironment found *in vivo*. Consequently, further research should involve more advanced experimental models, such as multicellular systems, co-cultures or three-dimensional models, as well as *in vivo* studies, which will enable a more comprehensive assessment of the mechanisms of action and the translational potential of the peptides under investigation.

## 5 Conclusion

CDP4 and UG28 are clearly potent proangiogenic peptides derived from CPNE7, acting on key stages of angiogenesis—from proliferation and migration to chemotaxis and the formation of stable, functional capillary structures. They directly activate endothelial cells and modulate the microenvironment by increasing the secretion of VEGF-D, EGF, and, to a lesser extent, HGF. CDP4 appears to play a supportive or synergistic role, but not a dominant one. Both peptides are biologically safe, non-cytotoxic and non-immunogenic, which is a significant advantage over classic VEGF/FGF-based therapies, which often cause side effects and disrupt vascular balance. The successful integration of UG 28 with the biomaterials further highlights their translational potential, supporting the further development of chitosan-based local administration or delivery systems, provided that the binding capacity of the substance, release kinetics and stability under physiological conditions are confirmed. Our findings provide the first evidence of the proangiogenic activity of CPNE7-derived peptides in human endothelial cells and identify them as highly promising candidates for further preclinical development.

## Data Availability

The original contributions presented in the study are included in the article/Supplementary Material, further inquiries can be directed to the corresponding authors.
