# Supplementary material for: CPNE7-derived peptides CDP4 and UG28 as pro-angiogenic factors for endothelial regeneration
Source: Front Pharmacol. 2026 Sep 7;17:1839788. doi: 10.3389/fphar.2026.1839788 (PMC13591009; doi:10.3389/fphar.2026.1839788)
Supplement: Supplementary file 1 [file Supplementaryfile1.docx]

**SUPPLEMENTARY**

***Results***

***Confirmation of ESCs phenotype***

***Cytometric analysis***

**Fig. S1.** The isolated cells showed high expression of endothelial-specific markers with a low percentage of negative markers, confirming their endothelial phenotype.

***Table S1. Fluorescently labeled antibodies and dyes (references)***

| CD 31 (PECAM-1) | APC-eFluor™ 780 | eBioscinece 47-0319-42 | RRID: AB_10730582 |
| --- | --- | --- | --- |
| CD 309 (VEGER-1, -2, /KDR/Flk-1) | Alexa Fluor 647 | BD 560494 | AB_1645503 |
| eNOS | Alexa Fluor 647 | BD 560102 | AB_1645410 |
| LDLR | PE | BD 565653 | AB_2739325 |
| CD 146 (MCAM, P1H12, S-endo-1) | Per CP 5.5 | BD 562134 | AB_10896319 |
| CD 133 (Prominin-1) | PE | BD 566593 | AB_2744281 |
| CD 14 (LPS-R) | ECD | Becman Coulter B92391 | AB_131278 |

Concentration used antibodies: 1:100

***Cytotoxicity assay***

LDH test (Fig. S2) was performed to assess the cytotoxicity of CDP4 and UG28 peptides toward ECs. None of the peptides induced a statistically significant increase in cytotoxicity relative to control cells. In both cases, a statistically significant increase in dehydrogenase was observed in cells treated with Triton X- 100 (positive control). Results are presented as percentage of cytotoxicity relative to positive controls (cells treated with Triton X-100).

**Fig. S2** Analysis of cytotoxicity of CDP derivatives (CDP4, UG28) against ECs after 48 h incubation. Graph shows mean ± SEM from 4 independent experiments (4 replicates in each, n=16), *- statistically significant differences compared to control (cells cultured in medium with 2% serum), Whitney's U-Mann test, p<0.05.

***Analysis of growth factor and cytokine secretion in culture supernatants***

The secretions of endothelial cells isolated from individual patients and treated with UG28 at a concentration of 10 μg/ml were evaluated. The graph in Fig. S3 shows the effect of the UG 28 peptide on the paracrine activity of endothelial cells. The highest values were recorded for the endothelial cells of patient 1 in the case of VEGF-C, VEGF-D, leptin, and HGF factors released into the supernatant. The cells of patient 2 responded to the peptide by secreting EGF and FGF-2 relative to the control. Patient 3 responded with the lowest secretion of almost all tested factors except VEGF-D. The effect of peptide stimulation on endothelial cells was highly variable and specific to the cells of individual patients, as each patient had individual paracrine properties of EC cells.

**Fig. S3** The graphs show the levels of individual cytokines and growth factors secreted into the culture medium from ECs after stimulation with UG28 peptide in individual patients. C - control without stimulation. The study was performed for 3 patients.

***Immunogenicity and allergenicity of peptides and chitosan***

Immunoassays showed no immunogenic effect of CDP4 and UG28 peptide and chitosan substrates on human Th and Tc cells.

Peptides did not induce PBMC activation or basophil degranulation, indicating the absence of an immune response. This is particularly important, as immunogenicity is one of the major limitations of therapies based on large proteins. These results confirm that CPNE7‑derived peptides are safe and can be incorporated into biomaterials without the risk of triggering inflammatory reactions.

**Fig. S4** Analysis of immunogenicity markers on Th and Tc lymphocytes - against CDP4 and UG28 peptides and chitosan substrate. C+ represented samples with IgE. Graph shows mean ±SEM of 4 (against peptides) and 2 (against chitosan) independent experiments, *- statistically significant differences compared to control, Whitney U-Mann test, p<0.05

A basophil activation test performed on blood samples from four volunteers showed basophil stimulation in only one patient in response to CDP4 peptide at a concentration of 25 µg/ml; the results for UG28 at concentrations of 10 and 25 µg/ml did not show basophil degranulation and were below the 5% threshold. The same test performed against chitosan substrates did not show basophil degranulation in the four patients tested.

**Fig. S5**. Activation of basophils after 15 min of incubation with CDP4 and UG28 peptides at two concentrations and with 75/500 chitosan and chitosan gel filtrate. Values represent the percentage of activated CRTH2+/CD203c+ basophils for individual patients (n=4) and median ±SEM. Threshold value for allergenic effect 5%.

***Assessment of functional differentiation of ECs on chitosan lyophilizate with a covalently conjugated peptide UG28***

ECs seeded on lyophilizate with attached UG28 were characterized by a coated, elongated cell structure (Fig.S6 C, D) with numerous protrusions (Fig. S6. E, F) that helped the cells to contact each other. The control image of the lyophilizate without the peptide (Fig. S6 B) did not reveal elongated cells and such numerous protrusions, which may indicate slower process of this type of structure formation, weaker cell-to-cell contact, and subsequent transmission of chemotactic signals between cells. Fig. S6 A shows a control image of the lyophilizate without ECs.

**Fig. S6** Photo shows a control image of lyophilizate without peptide and ECs (A). Control image of lyophilizate without peptide (Fig. B) 20 000x. ECs seeded on lyophilizate with attached UG28 (Figs. Cx10 000x, D 20 000x) with numerous protrusions (Figs. E10 000x, F40 000x). The arrows point to the coated endothelial cells.

Fig.S7 **Test XTT** - test Mann–Whitney U test with Holm correction confirmed significant differences (p-values adjusted using Holm's method; results consistent with Dunn's method).

The assumption of normality is not met; we use nonparametric tests.

| **Comparison test XTT** | **p (Holm) 48h** | **statistical  significance**  after 48h | **p (Holm) 72h** | **statistical  significance**  after 72h |
| --- | --- | --- | --- | --- |
| [**C vs FBS**](ca://s?q=Pokaz_wynik_C_vs_FBS) | <0.0001 | ******* | <0.0001 | *** |
| [**C vs CDP4‑10**](ca://s?q=Pokaz_wynik_C_vs_CDP4_10) | **ns** | **ns** | **0.014** | * |
| [**C vs UG28‑10**](ca://s?q=Pokaz_wynik_C_vs_UG28_10) | **ns** | **ns** | **0.019** | * |
| [**C vs CDP4‑25**](ca://s?q=Pokaz_wynik_C_vs_CDP4_25) | 0,01 | ***** | **0.006** | ** |
| [**C vs UG28‑25**](ca://s?q=Pokaz_wynik_C_vs_UG28_25) | 0,05 | ***** | **0.033** | * |
| [**FBS vs CDP4‑10**](ca://s?q=Pokaz_wynik_FBS_vs_CDP4_10) | <0.0001 | ******* | <0.0001 | *** |
| [**FBS vs UG28‑10**](ca://s?q=Pokaz_wynik_FBS_vs_UG28_10) | <0.0001 | ******* | <0.0001 | *** |
| [**FBS vs CDP4‑25**](ca://s?q=Pokaz_wynik_FBS_vs_CDP4_25) | <0.0001 | ******* | <0.0001 | *** |
| [**FBS vs UG28‑25**](ca://s?q=Pokaz_wynik_FBS_vs_UG28_25) | <0.0001 | ******* | <0.0001 | *** |
| [**CDP4‑10 vs UG28‑10**](ca://s?q=Pokaz_wynik_CDP4_10_vs_UG28_10) | **ns** |  | **0.041** | * |
| [**CDP4‑10 vs CDP4‑25**](ca://s?q=Pokaz_wynik_CDP4_10_vs_CDP4_25) | **ns** |  | **0.033** | * |
| [**UG28‑10 vs CDP4‑25**](ca://s?q=Pokaz_wynik_UG28_10_vs_CDP4_25) | **ns** |  | **0.049** | * |
| [**CDP4‑10 vs UG28‑25**](ca://s?q=Pokaz_wynik_CDP4_10_vs_UG28_25) | ns |  | ns | ns |
| [**UG28‑10 vs UG28‑25**](ca://s?q=Pokaz_wynik_UG28_10_vs_UG28_25) | ns |  | ns | ns |
| [**CDP4‑25 vs UG28‑25**](ca://s?q=Pokaz_wynik_CDP4_25_vs_UG28_25) | ns |  | ns | ns |

***p<0.001 — highly significant, **<0.01 — significant, *<0.05 — marginally significant, ns — not significant

Fig.S8 **Migration** - Mann-Whitney U test (p-values adjusted using Holm's method; results consistent with Dunn's method)

The assumption of normality is not met; we use nonparametric tests.

| **comparison** | **p (Holm)** | **statistical significance** |
| --- | --- | --- |
| **C vs CDP4‑10 mg** | 0.004 | ** |
| **C vs UG28‑10 mg** | 0.013 | * |
| **C vs CDP4‑25 mg** | <0.0001 | *** |
| **C vs UG28‑25 mg** | <0.0001 | *** |
| **CDP4‑10 mg vs UG28‑10 mg** | 0.19 | ns |
| **CDP4‑10 mg vs CDP4‑25 mg** | 0.002 | ** |
| **CDP4‑10 mg vs UG28‑25 mg** | <0.0001 | *** |
| **UG28‑10 mg vs CDP4‑25 mg** | 0.011 | * |
| **UG28‑10 mg vs UG28‑25 mg** | <0.0001 | *** |
| **CDP4‑25 mg vs UG28‑25 mg** | 0.003 | ** |

Fig.S9 **Chemotaxis** - Mann-Whitney U Test (p-values adjusted using Holm's method—results consistent with Dunn's test)

The assumption of normality is not met; we use nonparametric tests.

| **comparison** | **p (Holm)** | **statistical significance** |
| --- | --- | --- |
| **C vs FBS** | 0.021 | * |
| **C vs CDP4‑10 mg** | <0.0001 | *** |
| **C vs UG28‑10 mg** | 0.006 | ** |
| **C vs CDP4‑25 mg** | <0.0001 | *** |
| **C vs UG28‑25 mg** | <0.0001 | *** |
| **FBS vs CDP4‑10 mg** | <0.0001 | *** |
| **FBS vs UG28‑10 mg** | 0.014 | * |
| **FBS vs CDP4‑25 mg** | <0.0001 | *** |
| **FBS vs UG28‑25 mg** | <0.0001 | *** |
| **CDP4‑10 mg vs UG28‑10 mg** | 0.22 | ns |
| **CDP4‑10 mg vs CDP4‑25 mg** | 0.004 | ** |
| **CDP4‑10 mg vs UG28‑25 mg** | <0.0001 | *** |
| **UG28‑10 mg vs CDP4‑25 mg** | 0.012 | * |
| **UG28‑10 mg vs UG28‑25 mg** | <0.0001 | *** |
| **CDP4‑25 mg vs UG28‑25 mg** | 0.005 | ** |

Fig.S10 Representative HILIC chromatogram used to determine the degree of maleimidoglycine substitution of chitosan. Maleimidoglycine-functionalized chitosan was analyzed following acid hydrolysis. The peak highlighted by the green box (retention time: 14.132 min) corresponds to norleucine, which was used as the internal standard. The peak highlighted by the red box (retention time: 23.595 min) corresponds to glycine released from the maleimidoglycine linker during hydrolysis and used as the analytical marker of chitosan functionalization. Quantification of the glycine signal relative to the norleucine internal standard indicated a maleimidoglycine substitution degree of approximately 10%. This value describes the functionalization of chitosan with maleimidoglycine and does not represent a direct determination of CPLG-UG28 loading.
